# HPLC-Based Activity Profiling for Antiprotozoal Compounds in *Croton gratissimus* and *Cuscuta hyalina*

**DOI:** 10.3389/fphar.2020.01246

**Published:** 2020-08-14

**Authors:** Abdelhalim Babiker Mahmoud, Ombeline Danton, Marcel Kaiser, Sami Khalid, Matthias Hamburger, Pascal Mäser

**Affiliations:** ^1^ Parasite Chemotherapy Unit, Swiss Tropical and Public Health Institute, Basel, Switzerland; ^2^ Faculty of Science, University of Basel, Basel, Switzerland; ^3^ Faculty of Pharmacy, University of Khartoum, Khartoum, Sudan; ^4^ Faculty of Pharmacy, University of Science and Technology, Omdurman, Sudan

**Keywords:** *Croton gratissimus*, *Cuscuta hyalina*, antiprotozoal activity, HPLC activity profiling, flavonoids

## Abstract

In a screening of Sudanese medicinal plants for antiprotozoal activity, the chloroform fractions obtained by liquid-liquid partitioning from ethanolic extracts of fruits of *Croton gratissimus* var. *gratissimus* and stems of *Cuscuta hyalina* Roth ex Schult. exhibited *in vitro* activity against axenically grown *Leishmania donovani* amastigotes. This antileishmanial activity was localized by HPLC-based activity profiling. Targeted preparative isolation afforded flavonoids **1**–**6**, 3-methoxy-4-hydroxybenzoic acid (**7**), and benzyltetrahydroisoquinoline alkaloids laudanine (**8**) and laudanosine (**9**) from *C. gratissimus*, and pinoresinol (**10**), isorhamnetin (**11**), (-)-pseudosemiglabrin (**12**), and kaempferol (**13**) from *C. hyalina.* The antiprotozoal activity of **1**–**13** against *L. donovani* (axenic and intracellular amastigotes), *Trypanosoma brucei rhodesiense* (bloodstream forms), and *Plasmodium falciparum* (erythrocytic stages), and the cytotoxicity in L6 murine myoblast cells were determined *in vitro*. Quercetin-3,7-dimethylether (**6**) showed the highest activity against axenic *L. donovani* (IC_50,_ 4.5 µM; selectivity index [SI], 12.3), *P. falciparum* (IC_50,_ 7.3 µM; SI, 7.6), and *T. b. rhodesiense* (IC_50_, 2.4 µM; SI, 23.2). The congener ayanin (**2**) exhibited moderate antileishmanial (IC_50_, 8.2 µM; SI, 12.2), antiplasmodial (IC_50_, 7.8 µM; SI, 12.9), and antitrypanosomal activity (IC_50_, 11.2 µM; SI, 8.9). None of the compounds showed notable activity against the intramacrophage form of *L. donovani*.

## Introduction

Parasitic protozoa are the causative agents of devastating, yet often neglected diseases. The kinetoplastids, a group of flagellated protozoa, cause neglected tropical diseases that put more than one billion people around the globe at risk (WHO|World Health Organization, n.d.; Khalid, 2012). These diseases are human African trypanosomiasis (HAT) caused by *Trypanosoma brucei* spp., Chagas’ disease caused by *Trypanosoma cruzi*, and Leishmaniasis caused by *Leishmania* spp. (Stuart et al., 2008). The apicomplexan parasite *Plasmodium falciparum* is the causative agent of malaria tropica which claims more than 400,000 lives every year (Organization, W.H. 2019).

These infections are of high public health relevance and socio-economic impact. Most of the currently available drugs have drawbacks in terms of toxicity, limited availability of oral therapeutic dosage forms, development of resistance, or non-affordability.

Natural products have in many instances provided new leads to combat neglected tropical diseases (Schmidt et al., 2012). As part of an ongoing screening project of Sudanese medicinal plants for antiprotozoal activity (Mahmoud et al., 2020a; Mahmoud et al., 2020b), the chloroform extract of *Croton gratissimus* var. *gratissimus* (Euphorbiaceae), and *Cuscuta hyalina* Roth ex Schult. (Convolvulaceae) showed promising activity against *P. falciparum* and *Leishmania donovani*.

The genus *Croton* comprises over 1300 species that are widely distributed throughout tropical and subtropical regions of the world. *Croton* species have been used traditionally in Africa, South Asia, and Latin America for the treatment of infections and digestive disorders (Wu and Zhao, 2004; Xu et al., 2018). In Sudan, *C. gratissimus*, locally known as *Um-Geleigla*, has been used traditionally for the treatment of hypertension and malaria (Mohamed et al., 2009). The main secondary metabolites include flavonoids, terpenoids, and essential oil (Ngadjui et al., 2002; Aderogba et al., 2011; Yagi et al., 2016). Previous studies have demonstrated that the roots of *C. gratissimus* possessed antiplasmodial activity *in vivo* (Okokon and Nwafor, 2009). Cembranolide diterpenes isolated from the leaves were found to be active when tested against *P. falciparum* (Langat et al., 2011).

The genus *Cuscuta* comprises over 200 species distributed worldwide. They are stem obligate holoparasitic plants possessing neither roots nor fully expanded leaves. The interaction between parasite and host is established through haustoria (Kaiser et al., 2015). Different *Cuscuta* species have been used in traditional Indian and Chinese medicine. Cytotoxic, antioxidant, and antimicrobial activities have been reported (Ahmad et al., 2017). Previous phytochemical investigations of the genus *Cuscuta* identified flavonoids, lignans, alkaloids, fatty acids, and essential oil (Donnapee et al., 2014; Ahmad et al., 2017). The phytochemistry and antiparasitic activity of *C. hyalina* has not been studied.

In an earlier screening of Sudanese medicinal plants for antiprotozoal activity, the ethanolic extracts of *Croton gratissimus* fruits and *Cuscuta hyalina* stems had been found to exhibit *in vitro* antiprotozoal activity against axenic *L. donovani* (MHOM/ET/67/L82). Subsequent liquid- liquid partitioning against petroleum ether, chloroform, and ethyl acetate located the activity in the chloroform portion (Mahmoud et al., 2020b). We here report on the targeted isolation and structure elucidation of compounds responsible for the activity, and on their *in vitro* activity against *T. b. rhodesiense* (STIB 900), axenic and intramacrophage amastigotes of *L. donovani* (MHOM/ET/67/L82), and *P. falciparum* (NF54).

## Materials and Methods

### General Experimental Procedures

HPLC-grade methanol and acetonitrile from Macron Fine Chemicals (Avantor Performance Materials), and water from Milli-Q water purification systems (Merck Millipore) were used for HPLC separations. For fractionation and preparative separation, technical grade solvents from Scharlau (Scharlab S. L.) were used after distillation. Silica gel 60 F_254_ coated aluminum TLC plates were obtained from Merck. Silica gel (230–400 µm, Merck) and Sephadex LH-20 (25–100 µm, Sigma-Aldrich) were used for open column chromatography. Optical rotation was measured in methanol using a JASCO P-2000 digital polarimeter equipped with a sodium lamp (589 nm) and a temperature-controlled microcell (10 cm). UV and ECD spectra were recorded in methanol on a Chirascan CD spectrometer (Applied Photophysics) using 110 QS 1 mm path precision cells (Hellma Analytics). NMR spectra were recorded on a Bruker Avance III NMR spectrometer operating at 500.13 MHz for ^1^H and 125.77 MHz for ^13^C. ^1^H NMR, COSY, HSQC, HMBC, and NOESY spectra were measured at 23°C in a 1 mm TXI probe with a z-gradient, using standard Bruker pulse sequences. Spectra were analyzed by Bruker TopSpin 3.5 pl 7 and ACDLabs Spectrus Processor. NMR spectra were recorded in DMSO-d_6_ (99.9 atom % D; Armar Chemicals).

HPLC-PDA-ELSD-ESIMS data were recorded in positive- and negative-ion mode (scan range of *m/z* 200–1,500) on a Shimadzu LC-MS/MS 8030 triple quadrupole MS system, connected *via* a T-splitter (1:10) to a Shimadzu HPLC system consisting of degasser, binary mixing pump, autosampler, column oven, and a diode array detector and to an Alltech 3300 ELSD detector. Separation was achieved on a SunFire C_18_ (3.5 μm, 150 × 3.0 mm i.d.) column equipped with a guard column (10 mm × 3.0 mm i.d.) (Waters). Data acquisition and processing were performed with LabSolution software.

Microfractionation was carried out with the same HPLC instrument connected *via* a T split to an FC204 fraction collector (Gilson) with only UV detection, using a SunFire C_18_ (3.5 μm, 150 mm × 3.0 mm i.d.) column equipped with a guard column (10 mm × 3.0 mm i.d.) (Waters).

Semipreparative HPLC separations were carried out with an Agilent HP 1100 Series system consisting of a quaternary pump, autosampler, column oven, and a diode array detector. SunFire C_18_ (5 μm, 10 × 150 mm i.d.) columns (Waters) were used for separations. Chemstation software was used for data acquisition and processing. Preparative separations were carried out on a Puriflash 4100 system (Interchim) or a Reveleris PREP purification system (Büchi). Sephadex LH-20 (110 cm × 3 cm; 25–100 µm) and silica gel (40 cm × 5 cm, 230–400 mesh) columns were used.

All handling of infectious agents (*L. donovani*, *T. b. rhodesiense*, *P. falciparum*) was performed under strict biosafety level 2 conditions under notification A000275 to the Swiss Federal Office of Public Health.

### Plant Material

*Croton gratissimus* var. *gratissimus* fruits and *Cuscuta hyalina* Roth ex Schult. stems were obtained from the Herbarium of the Faculty of Pharmacy, University of Science and Technology, Omdurman, Sudan. The taxonomic identity was confirmed by the Medicinal and Aromatic Plants Research Institute, Sudan and voucher specimens (CZFCHL02 and ChSCHL 02) were deposited. Plant materials were dried at room temperature and milled before extraction.

### Extraction

Powdered materials of *C. gratissimus* fruits and *C. hyalina* stems (500 g each), respectively, were extracted with 1 L of 70% ethanol and kept in a magnet rod shaker for 24 h. The extraction procedure was repeated three times for each herbal drug. Extracts were filtered and dried under reduced pressure. For each plant, the ethanolic extract was suspended in water and partitioned successively with petroleum ether, chloroform, and ethyl acetate. Three repetitive partitioning procedures, each with 500 ml of either solvent were performed. This afforded 3.5 and 1.2 g of the chloroform extracts of *C. gratissimus* fruits and *C. hyalina* stems, respectively.

### Microfractionation

HPLC-based microfractionation of the chloroform extracts of *C. gratissimus* fruits and *C. hyalina* stems was performed [H_2_O + 0.1% formic acid (A), MeCN + 0.1% formic acid (B); 0→100% B (0–30 min), 100% B (30–40 min); flow rate 0.4 ml/min; sample concentration 10 mg/ml in DMSO; injection volume twice 35 μl] by collecting 1-min fractions from minute 1 to minute 40 into a 96-deepwell plate. After drying of plates in a Genevac EZ-2 evaporator, microfractions were tested for their antiprotozoal activity according to previously established protocols (Potterat and Hamburger, 2013; Potterat and Hamburger, 2014).

### Preparative Isolation

The chloroform fraction (3.5 g) of *C. gratissimus* fruits was fractionated by column chromatography (CC) on Sephadex LH-20 (110 × 3 cm; 25–100 µm) using methanol as eluent at a flow rate of 1 ml/min. A total of 19 fractions (A-S) were combined based on TLC patterns (silica gel; CH_2_CL_2_−MeOH, 90:10, 75:25, and 50:50, respectively; detection with 1% ethanolic vanillin and 10% sulfuric acid, followed by heating at 105°C). Fractions were submitted to HPLC-PDA-ELSD-MS analysis to track peaks previously detected in the active time windows of the activity profile.

Fraction M (36 mg) was submitted to semipreparative RP-HPLC [H_2_O (A), CH_3_CN (B); 43% B (0–22 min), 43→100% B (22–27 min), 100% B (27–30 min), flow rate 4 ml/min; sample concentration 50 mg/ml in DMSO; injection volume 50 μl], yielding quercetin-3,3′,4′-trimethylether (**1**, 0.3 mg, t_R_ 10.2 min), ayanin (**2**, 21.9 mg, t_R_ 16.7 min), and retusin (**3**, 0.4 mg, t_R_ 28.8 min).

Fraction O (15 mg) was submitted to semipreparative RP-HPLC [H_2_O (A), CH_3_CN (B); 35% B (0–34 min), 35→100% B (34–40 min), 100% B (40–45 min), flow rate 4 ml/min; sample concentration 50 mg/ml in DMSO; injection volume 50 μl], to afford naringenin (**4**, 0.51 mg, t_R_ 9.6 min), quercetin-3,4′-dimethylether (**5**, 1.9 mg, t_R_ 12.1 min), and quercetin-3,7-dimethylether (**6**, 7.1 mg, t_R_ 20.5 min).

Fraction K (26.6 mg) was purified by semipreparative RP-HPLC [H_2_O (A), CH_3_CN (B), both containing 0.1% formic acid; 10→32% B (0–30 min), 32→100% B (30–35 min), 100% B (35–40 min), flow rate 4 ml/min; sample concentration 50 mg/ml in DMSO; injection volume 50 μl], to afford 3-methoxy-4-hydroxybenzoic acid (**7**, 0.63 mg, t_R_ 10.9 min).

Fraction C (100.6 mg) was purified by semipreparative RP-HPLC [H_2_O (A), CH_3_CN (B), both containing 0.1% formic acid; 10→17% B (0–20 min), 17→100% B (20–25 min), 100% B (25–30 min), flow rate 4 ml/min; sample concentration 50 mg/ml in DMSO; injection volume 50 μl], to afford laudanine (**8**, 0.41 mg, t_R_ 9.1 min), and laudanosine (**9**, 0.63 mg, t_R_ 14.5 min).

The chloroform fraction (1.9 g) of *C. hyalina* stems was fractionated by CC on silica gel (40 × 5 cm, 230–400 mesh), using a gradient of CH_2_CL_2_−MeOH (99:1 to 0:100) as mobile phase. A total of 16 fractions (A-P) were combined based on TLC patterns (silica gel; CH_2_CL_2_−MeOH, 99:1, 90:10, and 80:20, respectively; detection with 1% ethanolic vanillin and 10% sulfuric acid, followed by heating). Fractions were submitted to HPLC-PDA-ELSD-MS analyses to track peaks previously detected in the active time windows of the activity profile.

Fraction B (52.7 mg) was purified by semipreparative RP-HPLC [H_2_O (A), CH_3_CN (B); 25→70% B (0–30 min), 70→100% B (30–33 min), 100% B (33–40 min), flow rate 4 ml/min; sample concentration 50 mg/ml in DMSO; injection volume 50 μl], to afford pinoresinol (**10**, 7.8 mg, t_R_ 10.2 min), isorhamnetin (**11**, 3.5 mg, t_R_ 13.6 min), pseudosemiglabrin (**12**, 2.2 mg, t_R_ 23.5 min). Kaempferol (**13**) was identified by co-injection of a reference standard (Sigma-Aldrich).

*Quercetin-3,3′,4′-trimethylether* (**1**): amorphous solid; ^1^H and ^13^C NMR, see **Table S1**,

Supporting Information; ESIMS *m/z* 345 [M + H]^+^.

*Ayanin* (**2**): amorphous solid; ^1^H and ^13^C NMR, see **Table S1**, Supporting Information; ESIMS *m/z* 345 [M + H]^+^.

*Retusin* (**3**): amorphous solid; ^1^H and ^13^C NMR, see **Table S1**, Supporting Information; ESIMS *m/z* 359 [M + H]^+^.

*Naringenin* (**4**): amorphous solid; ^1^H and ^13^C NMR, see **Table S2**, Supporting Information; ESIMS *m/z* 273 [M + H]^+^.

*Quercetin-3,4′-dimethylether* (**5**): amorphous solid; ^1^H and ^13^C NMR, see **Table S2**, Supporting Information; ESIMS *m/z* 331 [M + H]^+^.

*Quercetin-3,7-dimethylether* (**6**): amorphous solid; ^1^H and ^13^C NMR, see **Table S2**, Supporting Information; ESIMS *m/z* 331 [M + H]^+^.

*3-Methoxy-4-hydroxybenzoic acid* (**7**): amorphous solid; ^1^H and ^13^C NMR, see **Table S3**, Supporting Information; ESIMS *m/z* 169 [M + H]^+^.

*R-Laudanine* (**8**): amorphous solid; [α]^25^_D_ -6.6 (*c* 0.04, MeOH); UV λ_max_ (MeOH) (log ϵ) 226 (0.07), 291 (0.01) nm; ECD (MeOH, *c* 3.5 × 10^−4^ M, 1 mm path length) λ_max_(Δϵ) 214 (−0.56), 241 (−0.47), 290 (−0.39); ^1^H and ^13^C NMR, see **Table S4**, Supporting Information; ESIMS *m/z* 344 [M + H]^+^.

*R-Laudanosine* (**9**): amorphous solid; [α]^25^_D_ −62.5 (*c* 0.04, MeOH); UV λ_max_ (MeOH) (log ϵ) 201 (0.75), 226 (0.18), 279 (0.06) nm; ECD (MeOH, *c* 1.4 × 10^−4^ M, 1 mm path length) λ_max_(Δϵ) 211 (−19.43), 241 (−6.67), 290 (−3.38); ^1^H and ^13^C NMR, see **Table S4**, Supporting Information; ESIMS *m/z* 358 [M + H]^+^.

*(+)-(7S,7′S,8R,8′R)-Pinoresinol* (**10**): amorphous solid; [α]^25^_D_ 69.0 (*c* 0.10, MeOH); UV λ_max_ (MeOH) (log ϵ) 202 (0.70), 232 (0.10) nm; ECD (MeOH, *c* 7.0 × 10^−5^ M, 1 mm path length) λ_max_(Δϵ) 207 (+21.78) nm; ^1^H and ^13^C NMR, see **Table S5**, Supporting Information; ESIMS *m/z* 359 [M + H]^+^.

*Isorhamnetin* (**11**): amorphous solid; ^1^H and ^13^C NMR, see **Table S5**, Supporting Information; ESIMS *m/z* 317 [M + H]^+^.

*(-)-(3′′S,4′′R,5′′S)-Pseudosemiglabrin* (**12**): amorphous solid; [α]^25^_D_ −410.0 (*c* 0.05, MeOH); UV λ_max_ (MeOH) (log ϵ) 212 (0.73), 255 (0.49), 309 (0.43) nm; ECD (MeOH, *c* 2.6 × 10^−4^ M, 1 mm path length) λ_max_(Δϵ) 206 (−12.84), 215 (+3.69), 226 (−10.40), 257 (−11.01), 275 (−7.99) nm; ^1^H and ^13^C NMR, see **Table S6**, Supporting Information; ESIMS *m/z* 393 [M + H]^+^.

*Kaempferol* (**13**): identified by co-injection of a reference standard (Sigma-Aldrich).

### Sample Preparation

Compounds were dissolved in DMSO (10 mg/ml) and warmed up to 40°C and/or sonicated if necessary. These DMSO stocks were kept at −20°C. For each assay, a fresh dilution to 100 µg/ml in medium was prepared. This was used to prepare the serial dilutions directly in the 96-well assay plates. Since DMSO is cytotoxic, the maximum DMSO concentration in the test was 1%.

### Activity Against *Leishmania donovani* Axenic Amastigotes

Amastigotes of *L. donovani* strain MHOM/ET/67/L82 were grown under an atmosphere of 5% CO_2_ in air in axenic culture at 37°C in SM medium (Cunningham, 1977) at pH 5.4 supplemented with 10% heat-inactivated fetal bovine serum. 50 µL of culture medium was added in the wells of a 96-well plate and serial drug dilutions of eleven three-fold dilution steps covering a final range from 100 to 0.002 μg/ml were prepared. 50 µL culture medium with 2 × 10^5^ amastigotes from axenic culture were added to each well. After 70 h of incubation the plates were inspected under an inverted microscope to assure growth of the controls and sterile conditions. 10 μl of resazurin (12.5 mg resazurin dissolved in 100 ml distilled water) were added to each well and the plates incubated for another 2 h. Then the plates were read with a Spectramax Gemini XS microplate fluorometer (Molecular Devices Cooperation, Sunnyvale, CA, USA) using an excitation wavelength of 536 nm and an emission wavelength of 588 nm. Data were analyzed using the software Softmax Pro (Molecular Devices Cooperation, Sunnyvale, CA, USA). Decrease of fluorescence (= inhibition) was expressed as percentage of the fluorescence of untreated control cultures and plotted against the drug concentrations. From the sigmoidal inhibition curves the IC_50_ values were calculated. Miltefosine was used as positive control drug. Assays were performed in two independent replicates at least.

### Activity Against *Leishmania donovani* Intramacrophage Amastigotes

Macrophages were isolated from the mouse (CDI) peritoneal cavity (Zhang et al., 2008) using 2% starch solution as eliciting agent injected 2 day prior to cell harvest. Animal work was carried out according to the rules and regulations for the protection of animal rights (“Tierschutzverordnung”) of the Swiss “Bundesamt für Veterinärwesen” (License number 2374). Mouse peritoneal macrophages (4 × 10^4^ in 100 µL RPMI 1640 medium with 10% heat-inactivated FBS) were seeded into wells of a 96-well plate. After 24 h, 2 × 10^5^ amastigote *L. donovani* in 100 µL were added. The amastigotes were taken from an axenic amastigote culture grown at pH 5.4. The medium containing free amastigote forms was removed after 24 h and replaced with fresh medium. The washing step was repeated and afterward the serial drug dilution was prepared with at least 6 dilution steps. Compounds were dissolved in DMSO at 10 mg/ml and further diluted in medium. After 96 h of incubation at 37°C under a 5% CO_2_ atmosphere, the medium was removed, and cells were fixed by adding 50 µl 4% formaldehyde solution followed by a staining with a 5 µM DRAQ5 solution. Plates were imaged in ImageXpress XLS (MD) microscope using a 20× air objective (635 nm excitation: 690/50 emission). 9 images were collected per well. Automated image analysis was performed with a script developed on Meta Xpress Software (MD). Three outputs were provided for each well: i) number of host cell nuclei; ii) numbers of infected and non-infected host cells; iii) number of parasite nuclei per infected host cell. The IC_50_ values were calculated based on the infection rate and the numbers of intracellular amastigotes. The cytotoxicity to macrophages was determined in parallel, and IC_50_ values were calculated based on the numbers of surviving, uninfected macrophages. Miltefosine was used as control. Assays were performed in two independent replicates at least.

### Activity Against *Trypanosoma brucei rhodesiense* STIB900

The stock was originally isolated from a Tanzanian patient and adapted to axenic culture conditions after several mouse passages and cloned. Minimum Essential Medium (50 µl) supplemented with 25 mM HEPES, 1 g/L additional glucose, 1% MEM non-essential amino acids (100×), 0.2 mM 2-mercaptoethanol, 1 mM Na-pyruvate (Baltz et al., 1985) and 15% heat inactivated horse serum was added to each well of a 96-well microtiter plate. Serial drug dilutions of eleven 3-fold dilution steps covering a range from 100 to 0.002 μg/ml were prepared. Then 4 × 10^3^ bloodstream forms of *T. b. rhodesiense* STIB 900 in 50 µL were added to each well and the plate incubated for 70 h at 37°C and under a 5% CO_2_ atmosphere. 10 µL resazurin solution (resazurin, 12.5 mg in 100 ml double-distilled water) was then added to each well and incubation continued for a further 2–4 h (Räz et al., 1997). Plates were read with a Spectramax Gemini XS microplate fluorometer (Molecular Devices Cooperation, Sunnyvale, CA, USA) using an excitation wavelength of 536 nm and an emission wavelength of 588 nm. Softmax Pro program (Molecular Devices Cooperation, Sunnyvale, CA, USA) was used for data analyses and IC_50_ values were calculated by linear regression (Huber and Koella, 1993), and 4-parameter logistic regression from the sigmoidal dose inhibition curves. Melarsoprol (Arsobal Sanofi-Aventis, received from WHO) was used as control. Assays were performed in two independent replicates at least.

### Activity Against *Plasmodium falciparum*

*In vitro* activity against the erythrocytic stages of *P. falciparum* was determined using a ^3^H-hypoxanthine incorporation assay (Desjardins et al., 1979), using the drug sensitive NF54 strain (Ponnudurai et al., 1981). Compounds were dissolved in DMSO at 10 mg/ml and further diluted in medium before addition to parasite cultures incubated in RPMI 1640 medium without hypoxanthine, supplemented with HEPES (5.94 g/L), NaHCO_3_ (2.1 g/L), neomycin (100 U/ml), Albumax^R^ (5 g/L), and washed human red cells A^+^ at 2.5% haematocrit (0.3% parasitemia). Serial drug dilutions of 11 three-fold dilution steps covering a range from 100 to 0.002 μg/ml were prepared. The 96-well plates were incubated in a humidified atmosphere at 37°C; 4% CO_2_, 3% O_2_, 93% N_2_. After 48 h, 50 μl of ^3^H-hypoxanthine (=0.5 μCi) was added to each well of the plate. The plates were incubated for a further 24 h under the same conditions. The plates were then harvested with a Betaplate™ cell harvester (Wallac, Zurich, Switzerland), the red blood cells transferred onto a glass fibre filter, and lysed with distilled water. The dried filters were inserted into a plastic foil with 10 ml of scintillation fluid and counted in a Betaplate™ liquid scintillation counter (Wallac, Zurich, Switzerland). IC_50_ values were calculated from sigmoidal inhibition curves by linear regression using Microsoft Excel. Chloroquine (Sigma C6628) was used as control. Assays were performed in two independent replicates at least.

### *In Vitro* Cytotoxicity With L-6 Cells

Assays were performed in 96-well microtiter plates, each well containing 100 µl of RPMI 1640 medium supplemented with 1% l-glutamine (200 mM) and 10% fetal bovine serum, and 4000 L-6 cells (a primary cell line derived from rat skeletal myoblasts) (Ahmed et al., 1994). Serial drug dilutions of 11 three-fold dilution steps covering a range from 100 to 0.002 μg/ml were prepared 24 h post seeding L-6 cells. The plates were incubated for 70 h and inspected under an inverted microscope to assure growth of the controls and sterile conditions. 10 µl of resazurin was then added to each well and the plates incubated for another 2 h. Then the plates were read with a Spectramax Gemini XS microplate fluorometer (Molecular Devices Cooperation, Sunnyvale, CA, USA) using an excitation wavelength of 536 nm and an emission wavelength of 588 nm. The IC_50_ values were calculated by linear regression and four-parameter logistic regression from the sigmoidal dose inhibition curves using SoftmaxPro software (Molecular Devices Cooperation, Sunnyvale, CA, USA). Podophyllotoxin (Sigma P4405) was used as positive control. All assays were performed in two independent replicates at least. Activities of all compounds were expressed in µM using the formula:

Activity (μM)=Activity (μg/ml)×1000/Molecular weight.

## Results and Discussion

### Extraction and HPLC-Based Activity Profiling

**Figure 1:**
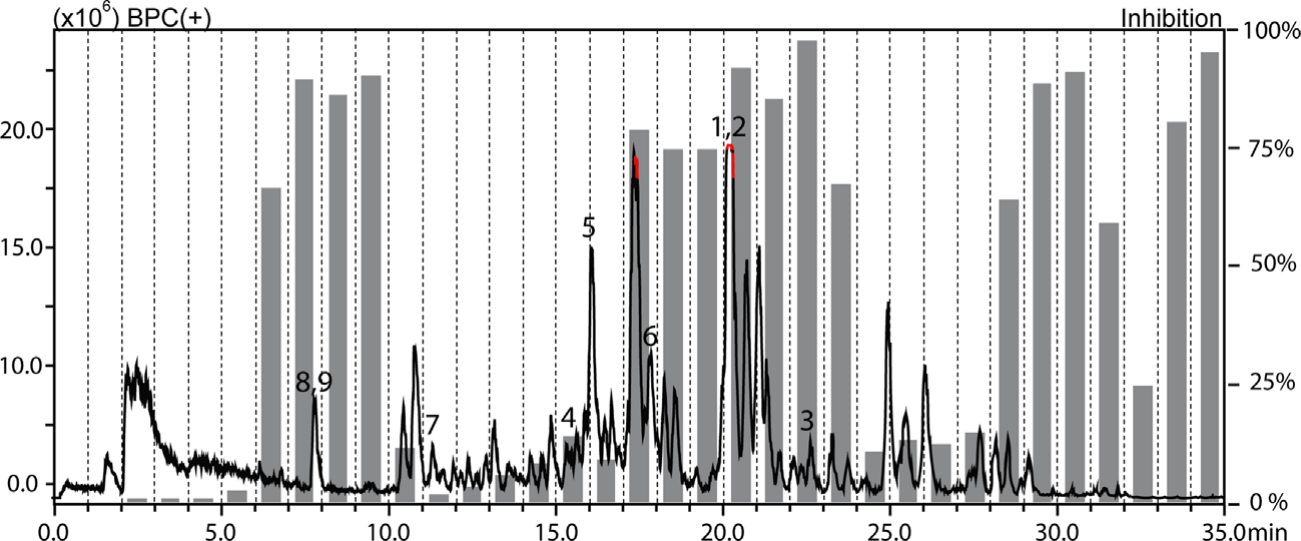
HPLC-based activity profiling of the chloroform fraction of *Croton gratissimus* var. *gratissimus* against axenic amastigotes of *L. donovani*. The ESIMS (positive base peak chromatogram) of a separation of 300 μg of fraction on an analytical RP-HPLC column is shown. Activities of 1-min microfractions are shown with grey columns, and are expressed as percent growth inhibition compared to untreated parasites. Bold numbers in the chromatogram refer to compounds **1**–**9**

**Figure 2:**
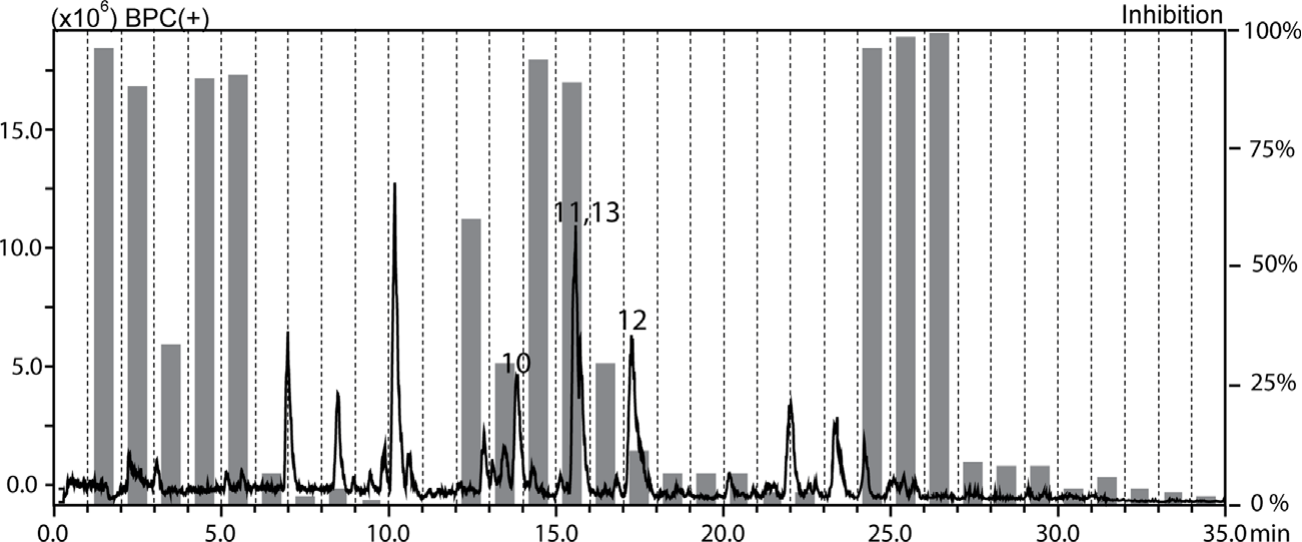
HPLC-based activity profiling of the chloroform fraction of *Cuscuta hyalina* Roth ex Schult. against axenic amastigotes of *L. donovani*. The ESIMS (positive base peak chromatogram) and activity profile (gray bars) are shown. Bold numbers in the chromatogram refer to compounds **10**–**13**.

The methanolic extracts of *Croton gratissimus* var. *gratissimus* fruits and *Cuscuta hyalina* Roth ex Schult. stems had been previously found to exhibit antiprotozoal activity (Mahmoud et al., 2020b). The antileishmanial activity displayed by the chloroform fractions of the two plants was tracked by HPLC-based activity profiling, a procedure combining analytical separation with on-line spectroscopy and time-based microfractionation for bioactivity testing (Potterat and Hamburger, 2013; Potterat and Hamburger, 2014). One-minute microfractions were collected and tested for *L. donovani* growth inhibition. The HPLC−ESIMS (positive base peak chromatograms) trace and the corresponding antileishmanial activity profiles for *C. gratissimus* and *C. hyalina* are shown in **Figures 1** and **2**. Major antileishmanial activity and a series of distinct peaks in the HPLC-ESIMS trace were observed in the time window between 18 and 24 min for *C. gratissimus*, and between 13 and 17 min for *C. hyalina*.

### Compound Isolation and Structure Elucidation

Separation of the chloroform fraction of *C. gratissimus* on a Sephadex LH-20 column yielded 19 subfractions (A-S). Based on the HPLC−PDA-ESIMS analysis, subfractions M, O, K, and C were found to contain peaks associated with the active time window. Further purification by semipreparative RP-HPLC afforded compounds **1**–**3** from subfraction M, **4**–**6** from subfraction O, **7** from subfraction K, and **8** and **9** from subfraction C.

By means of 1D and 2D NMR data (**Tables S1**-**S4**; Supporting Information), six flavonoids were identified as quercetin-3,3′,4′-trimethylether (**1**) (Urbatsch et al., 1976), ayanin (**2**) (Matsuda et al., 2002), retusin (**3**) (Matsuda et al., 2002), naringenin (**4**) (Ibrahim et al., 2003), quercetin-3,4′-dimethyl ether (**5**) (Barberá et al., 1986a), quercetin-3,7-dimethylether (**6**) (Wang et al., 1989), along with 3-methoxy-4-hydroxybenzoic acid (**7**) (Crestini et al., 2006), and the two benzyltetrahydroisoquinoline alkaloids laudanine (**8**) (Janssen et al., 1990) and laudanosine (**9**) (Janssen et al., 1990). For naringenin (**4**), an optical rotation close to 0 and the absence of a Cotton effect (CE) in the ECD indicated a 1:1 mixture of *R*- and *S*-stereoisomers. The absolute configuration of **8 ** and **9** was determined as *R* based on the optical rotation ([α]^25^_D_ -6.6 (*c* 0.04, MeOH) for **8** (Frydman et al., 1958) and [α]^25^_D_ -62.5 (*c* 0.04, MeOH) for **9** (Ruiz-Olalla et al., 2015)). Moreover, the ECD spectra of both compounds showed two negative cotton effects (CEs) at 210 to 215 nm and 240 to 242 nm which were in good agreement with calculated spectra of the *R*-stereoisomers (**Figure S1** and **S2**, Supporting Information).

**Figure 3:**
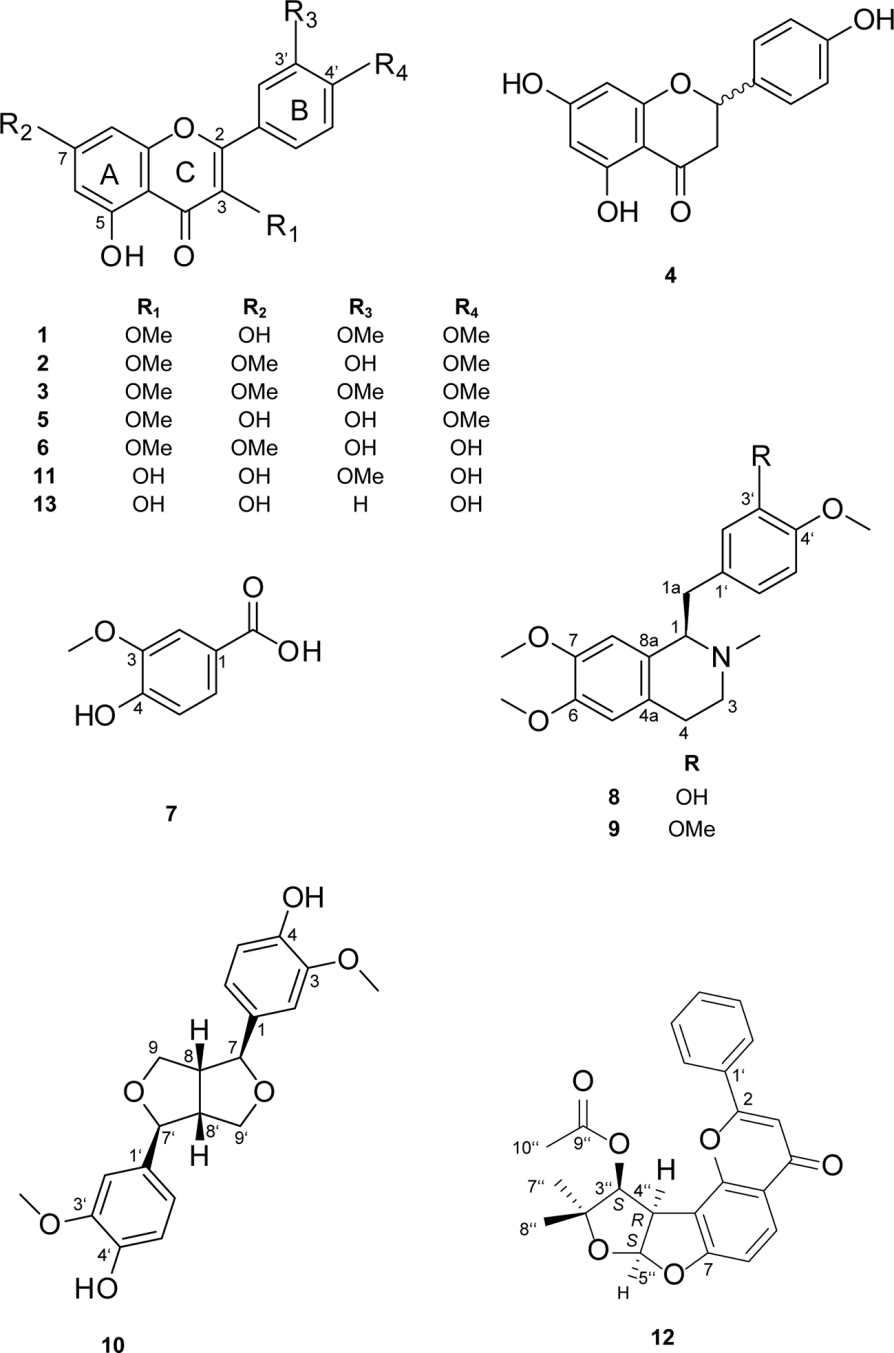
Chemical structures of compounds **1**–**13**.

Compounds **1**–**9** (**Figure 3**) are reported here for the first time from *C.* *gratissimus*, but some have been previously identified in other *Croton* species, such as ayanin (**2**) and quercetin-3,7-dimethylether (**6**) from *C. schiedeanus* (Guerrero et al., 2002), quercetin-3,4′-dimethylether (**5**) from *C. arboreus* (Aguilar-Guadarrama and Rios, 2004), 3-methoxy-4-hydroxybenzoic acid (**7**) from *C. tonkinensis* (Kuo et al., 2013), and *R*-laudanine (**8**) and *R*-laudanosine (**9**) from leaves and stems of *C. celtidifolius* (Amaral and Barnes, 1997).

Preparative chromatography on silica gel of the chloroform fraction of *C. hyalina* yielded 16 subfractions (A-P). Peaks associated with the active time window were detected in subfraction B. Further separation by semipreparative RP-HPLC afforded compounds **10**–**12** (**Figure 3**).

Based on the NMR data (**Tables S5** and **S6**, Supporting Information), compounds were identified as the lignan pinoresinol (**10**) (Abe and Yamauchi, 1988) and as flavonoids isorhamnetin (**11**) (Barberá et al., 1986b) and (-)-pseudosemiglabrin (**12**) (Waterman and Khalid, 1980). In addition, kaempferol (**13**) was identified by dereplication with a reference compound. The absolute configuration of **10** and **12** was established based on their optical activity and ECD spectra. For compound **10**, the optical rotation [α]^25^_D_ + 69.0 (*c* 0.10, MeOH) and the positive cotton effect at 207 nm (Δϵ +21.78) in the ECD spectrum indicated a (+)-(7*S*,7*′S*,8*R*,8*′R)* configuration of pinoresinol (**Figure S3**, Supporting Information). The optical rotation [α]^25^_D_ -410.0 (*c* 0.05, MeOH) of **12** indicated (-)-pseudosemiglabrin. The ECD spectrum showed four negative CEs at 206 (Δϵ -12.84), 226 (Δϵ -10.40), 257 (Δϵ -11.01), 275 (Δϵ -7.99) nm, and a positive CE at 215 nm (Δϵ +3.69). This was in agreement with calculated spectra for the 3′′*S*,4′′*R*,5′′*S* stereoisomer (**Figure S4**, Supporting Information), and opposite to the ECD data published for (+)-pseudosemiglabrin (Pirrung and Lee, 1995). However, the assignment of C-5′′ as *S* by Pirrung and Lee was incorrect. The absolute configuration of (-)-pseudosemiglabrin (**12**) was thus assigned as 3*′′S*,4*′′R*,5*′′S.*

Kaempferol (**13**) and isorhamnetin (**11**) have been previously reported from different *Cuscuta* species (Ahmad et al., 2017), while pinoresinol has been identified in *C. chinensis* (Yahara et al., 1994). To the best of our knowledge, this is the first report on isolation of pseudosemiglabrin (**12**) from *Cuscuta* species.

### Activity Against *Leishmania donovani* Axenic and Intracellular Amastigotes

All compounds were tested for their activity against *L. donovani* (MHOM/ET/67/L82) axenic amastigotes (**Table 1**). Quercetin-3,7-dimethylether (**6**) has shown the highest activity (IC_50_ 4.5 ± 0.3 µM), followed by ayanin (**2**) (IC_50_ 8.2 ± 1.6 µM). Both compounds exhibited similar selectivity indices (SI 12.3), which were the highest among the tested compounds.

**Table 1:** *In vitro* activity of compounds **1**–**13** against *T. b. rhodesiense* (STIB 900), *L. donovani* (MHOM-ET-67/L82) axenic and intracellular amastigotes, *P. falciparum* (NF54), and cytotoxicity in L6 cells and intramacrophages.

		IC_50_ *^a^* (µM)					
No.	Compound	*L. donovani*		*T. b. rhodesiense*	*P. falciparum*	L6 cells	Cytotoxicity
		Axenic	Intramacrophage				Intramacrophage
**1**	Quercetin-3,3*′*,4*′*-trimethylether	18.8 ± 4.0 (9.8)*^b^*	>79.9	55.1 ± 13.2 (3.4)*^b^*	42.1 ± 12.5 (4.4)*^b^*	185.2 ± 15.4	154.5 ± 19.3
**2**	Ayanin	8.2 ± 1.6 (12.2)*^b^*	>95.9	11.2 ± 0.2 (8.9)*^b^*	7.8 ± 0.9 (12.9)*^b^*	100.3 ± 9.3	127.9 ± 3.5
**3**	Retusin	34.1 ± 4.7 (6.3)*^b^*	>185.8	83.8 ± 7.5 (2.6)*^b^*	23.4 ± 3.8 (9.2)*^b^*	215.1 ± 4.7	173.2 ± 0.0
**4**	Naringenin	41.8 ± 9.3 (5.6)*^b^*	>121.3	184.2 ± 9.9 (1.3)*^b^*	73.2 ± 5.9 (3.2)*^b^*	233.3 ± 36.9	332.9 ± 18.2
**5**	Quercetin-3,4*′*-dimethylether	15.2 ± 3.1 (1.3)*^b^*	>33.3	6.6 ± 3.2 (2.9)*^b^*	18.2 ± 6.7 (1.1)*^b^*	19.2 ± 1.0	42.7 ± 12.1
**6**	Quercetin-3,7-dimethylether	4.5 ± 0.3 (12.3)*^b^*	>66.7	2.4 ± 0.5 (23.2)*^b^*	7.3 ± 0.6 (7.6)*^b^*	55.4 ± 34.3	101.5 ± 49.7
**7**	3-Methoxy-4-hydroxybenzoic acid	153.3 ± 45.5 (3.9)*^b^*	>595.2	38.6 ± 5.9 (15.4)*^b^*	138.4 ± 12.2 (4.3)*^b^*	595.2 ± 0.0	>500
**8**	Laudanine	193.3 ± 0.0 (1.5)*^b^*	>291.5	143.1 ± 0.0 (2.0)*^b^*	78.6 ± 0.4 (3.7)*^b^*	291.5 ± 0.0	>200
**9**	Laudanosine	185.4 ± 0.0 (1.5)*^b^*	>186.3	101.5 ± 38.8 (2.8)*^b^*	76.1 ± 21.6 (3.7)*^b^*	280.1 ± 0.0	>245
**10**	Pinoresinol	151.4 ± 22.6 (1.6)*^b^*	>279.3	116.8 ± 0.3 (2.1)*^b^*	>139.7	241.9 ± 21.2	>270
**11**	Isorhamnetin	21.7 ± 7.9 (6.0)*^b^*	>210.4	25.9 ± 5.4 (5.0)*^b^*	21.6 ± 0.7 (6.0)*^b^*	130.2 ± 58.7	>198.9
**12**	Pseudosemiglabrin	18.9 ± 2.6 (4.1)*^b^*	>84.2	37.5 ± 6.1 (2.1)*^b^*	16.3 ± 2.7 (4.8)*^b^*	78.1 ± 29.8	144.6 ± 10.5
**13**	Kaempferol	20.8 ± 1.7 (5.3)*^b^*	>115.4	36.4 ± 8.7 (3.1)*^b^*	53.1 ± 7.7 (2.1)*^b^*	111.4 ± 16.6	151.0 ± 18.8
	Positive control	0.5 ± 0.2*^c^*	6.6 ± 0.3*^c^*	0.01 ± 0.003*^d^*	0.01 ± 0.002*^e^*	0.03 ± 0.005*^f^*	>24.1

^a^The IC_50_s are mean values from at least two independent replicates ± absolute deviation.

^b^Selectivity index (SI): IC_50_ in L6 cells divided by IC_50_ in the titled parasitic strain.

^c^Miltefosine, ^d^Melarsoprol, ^e^Chloroquine ^f^Podophyllotoxin.

Compounds **1** and **5** from *C. gratissimus*, and **11**–**13** from *C. hyalina* exhibited IC_50_ values in the range of 15 to 22 µM against axenic amastigotes. These compounds showed varying degrees of cytotoxicity in L6 rat skeletal cells. Moderate selectivity (selectivity indices (SI) of 4–6) toward *L. donovani* axenic amastigotes was observed for compounds **11**–**13**, while quercetin-3,4′-dimethylether (**5**) showed the highest toxicity (SI 1.3).

The benzyltetrahydroisoquinoline alkaloids laudanine (**8**) and laudanosine (**9**), and the furofurano lignan pinoresinol (**10**) showed only marginal activity against *L. donovani* axenic amastigotes (IC_50_ ˃ 150 µM). The weak activity of the alkaloids was in agreement with previous reports (García Díaz et al., 2019).

After a first testing against axenic amastigotes, compounds were tested against *L. donovani* amastigotes in mouse macrophages. However, in this more elaborate and more physiological model none of the compounds showed activity (**Table 1**). In general, IC_50_ values for the intramacrophage form are higher than those for the axenic amastigotes (Berry et al., 2018). This loss of activity in the intracellular model could be due to poor cellular permeability of the compounds, binding to cytosolic proteins in the host cell, or metabolism in the host cell phagolysosome (Burchmore and Barrett, 2001; De Rycker et al., 2013; Berry et al., 2018).

### Activity Against *Trypanosoma brucei rhodesiense*

All compounds were also tested for their *in vitro* activity against the blood stream form of *T. brucei rhodesiense* (STIB 900) (**Table 1**). As for *L. donovani*, quercetin-3,7-dimethylether (**6**) was the most active (IC_50_ 2.4 ± 0.5 µM) and most selective (SI 23.2). Quercetin-3,4*′*-dimethyl ether (**5**) was also active (IC_50_ 6.6 ± 3.2 µM) but had a low selectivity (SI 2.9). Ayanin (**2**) had an IC_50_ of 11.2 ± 0.2 µM with moderate cytotoxicity (SI 8.9), and isorhamnetin (**11**) an IC_50_ of 25.9 ± 5.4 µM and a SI of 5.0. Kaempferol (**13**), pseudosemiglabrin (**12**) and 3-methoxy-4-hydroxybenzoic acid (**7**) showed marginal activity (IC_50_ 36–39 µM), and IC_50_ ˃ 80 µM were determined for retusin (**3**), naringenin (**4**), laudanine (**8**), laudanosine (**9**) and pinoresinol (**10**).

### Activity Against *Plasmodium falciparum*

*In vitro* activity against the erythrocytic stages of the *P. falciparum* drug sensitive strain NF54 was determined for all compounds (**Table 1**). Ayanin (**2**) and quercetin-3,7-dimethylether (**6**) showed IC_50_ values of 7 to 8 µM, but **2** exhibited a higher selectivity index (SI 12.9) than **6** (SI 7.6). Quercetin-3,4*′*-dimethylether (**5**) was also rather active (IC_50_ <20 µM) but equally showed cytotoxic in L6 cells. Isorhamnetin (**11**) and pseudosemiglabrin (**12**) showed IC_50_ around 20 µM against *P. falciparum* and a moderate degree of selectivity towards the parasite (SI ~ 5). 3-Methoxy-4-hydroxybenzoic acid (**7**), and pinoresinol (**10**) were the least active among the tested compounds.

### Correlation Between Chemical Structure of Isolated Flavonoids and Antiprotozoal Activity

Of the isolated compounds, only flavones showed notable activity (**Table 1**). From a comparison of flavones **1**–**3**, **5**, **6**, **10,** and **13** the following conclusions can be drawn: Compounds with a hydroxyl group at C-3*′* (**2**, **5**, and **6**) were the most active against the three parasites, whereby a catechol moiety as in **6** further increased the activity. Free hydroxyl groups at C-3 or C-7 (as in **5**, **11,** and **13**) had not result in significant *in vitro* activity. Compounds **2** and **6** exhibited the highest selectivity, while **5** showed significant cytotoxicity in L6 cells leading to a low SI.

Naringenin (**4**) displayed the weakest antiparasitic activity among the tested flavonoids. The presence of a double bond between C-2 and C-3 has been previously found to be essential for antiparasitic activity (Tasdemir et al., 2006). Overall, our results were in agreement with previous structure-activity studies of flavonoids (Tasdemir et al., 2006).

The influence of a balance between antioxidant and prooxidant properties of flavonoids on antiparasitic activity, and a correlation with their chemical structure has been investigated with the aid of QSAR models (Baldim et al., 2017). Compounds that displayed moderate to higher antitrypanosomal activity shared structural features, such as Δ^2,3^ unsaturation, presence of a hydroxyl group at C-3, a carbonyl group at C-4, and a catechol moiety in ring B. Our results were in line with these findings. To the best of our knowledge, the antitrypanosomal activities of quercetin-3,7-dimethylether (**6**) and ayanin (**2**) are here reported for the first time.

## Data Availability Statement

The raw data supporting the conclusions of this article will be made available by the authors, without undue reservation.

## Author Contributions

Conceived and designed the experiments: AM, MK, MH. Performed the experiments: AM, OD, MK. Analyzed the data: OD, PM, MH, SK. Wrote the paper: AM, OD, PM, MK, MH, SK.

## Funding

This work was supported by grants to AM by the Amt für Ausbildungsbeiträge Basel (www.hochschulen.bs.ch/ueber-uns/organisation/amtausbildungsbeitraege.html) and the Emilia Guggenheim-Schnurr Foundation (www.ngib.ch/stiftung-egs). The funders had no role in study design, data collection and analysis, decision to publish, or preparation of the manuscript.

## Conflict of Interest

The authors declare that the research was conducted in the absence of any commercial or financial relationships that could be construed as a potential conflict of interest.
