# Supplementary material for: HPLC-Based Activity Profiling for Antiprotozoal Compounds in Croton gratissimus and Cuscuta hyalina
Source: Front Pharmacol. 2020 Aug 14;11:1246. doi: 10.3389/fphar.2020.01246 (PMC7456963; doi:10.3389/fphar.2020.01246)
Supplement: Supplementary file 1 [file DataSheet_1.docx]

**SUPPORTING INFORMATION:**

**HPLC-Based Activity Profiling for Antiprotozoal Compounds in *Croton gratissimus* and *Cuscuta hyalina***

**Abdelhalim Babiker Mahmoud^1,2,3^, Ombeline Danton^2^, Marcel Kaiser^1,2^, Sami Khalid^3,4^, Matthias Hamburger^2^, and Pascal Mäser^1,2^**

^1^ Swiss Tropical and Public Health Institute, Basel, Switzerland

^2^ University of Basel, Basel, Switzerland

^3^ Faculty of Pharmacy, University of Khartoum, Khartoum, Sudan

^4^ Faculty of Pharmacy, University of Science and Technology, Omdurman, Sudan

Table of Contents

**Table S 1.** ^1^H and ^13^C NMR Spectroscopic Data for Compounds **1**-**3** (DMSO-*d6*; 500.13 Hz for ^1^H and 125.77 for ^13^C NMR; δ in ppm) **1**

**Table S 2.** ^1^H and ^13^C NMR Spectroscopic Data for Compounds **4**-**6** (DMSO-*d6*; 500.13 Hz for ^1^H and 125.77 for ^13^C NMR; δ in ppm) **2**

**Table S 3.** ^1^H and ^13^C NMR Spectroscopic Data for Compound **7** (DMSO-*d6*; 500.13 Hz for ^1^H and 125.77 for ^13^C NMR; δ in ppm) **3**

**Table S 4.** ^1^H and ^13^C NMR Spectroscopic Data for Compounds **8** and **9** (DMSO-*d6*; 500.13 Hz for ^1^H and 125.77 for ^13^C NMR; δ in ppm) **4**

**Table S 5.** ^1^H and ^13^C NMR Spectroscopic Data for Compounds **10** and **11** (DMSO-*d6*; 500.13 Hz for ^1^H and 125.77 for ^13^C NMR; δ in ppm) **5**

**Table S 6.** ^1^H and ^13^C NMR Spectroscopic Data for Compound **12** (DMSO-*d6*; 500.13 Hz for ^1^H and 125.77 for ^13^C NMR; δ in ppm) **6**

**Figure S 1.** Comparison of experimental and calculated UV (A) and ECD (B) spectra for compound **8** in MeOH (0.12 mg/mL). **7**

**Figure S 2.** Comparison of experimental and calculated UV (A) and ECD (B) spectra for compound **9** in MeOH (0.05 mg/mL). **8**

**Figure S 3.** Comparison of experimental and calculated UV (A) and ECD (B) spectra for compound **10** in MeOH (0.025 mg/mL). **9**

**Figure S 4.** Comparison of experimental and calculated UV (A) and ECD (B) spectra for compound **12** in MeOH (0.1 mg/mL). **10**

Computational Methods for ECD calculation 11

Table S 1. ^1^H and ^13^C NMR Spectroscopic Data for Compounds 1-3 (DMSO-*d6*; 500.13 Hz for ^1^H and 125.77 for ^13^C NMR; δ in ppm)

|  |  | **1** | |  | **2** | |  | **3** | |
| --- | --- | --- | --- | --- | --- | --- | --- | --- | --- |
| Position |  | δ_C_*^a^* | δ_H_ (mult *J* in Hz*)* |  | δ_C_*^a^* | δ_H_ (mult *J* in Hz*)* |  | δ_C_*^a^* | δ_H_ (mult *J* in Hz*)* |
| 2 |  | C*^b^* |  |  | 155.2, C |  |  | 155.4, C |  |
| 3 |  | 137.4, C |  |  | 137.9, C |  |  | 138.3, C |  |
| 4 |  | C*^b^* |  |  | 177.8, C |  |  | C^b^ |  |
| 5 |  | 160.9, C |  |  | 160.8, C |  |  | 161.0, C |  |
| 6 |  | 101.4, CH | 5.73, d (1.5) |  | 97.2, CH | 6.20, br s |  | 97.8, CH | 6.36, br s |
| 7 |  | C*^b^* |  |  | 164.8, C |  |  | 165.2, C |  |
| 8 |  | 95.3, CH | 5.91, d (1.2) |  | 91.7, CH | 6.47, br s |  | 92.4, CH | 6.74, br s |
| 9 |  | 157.2, C |  |  | 155.9, C |  |  | 156.3, C |  |
| 10 |  | 100.4, C |  |  | 105.0, C |  |  | 105.3, C |  |
| 1' |  | 123.1, C |  |  | 122.2, C |  |  | C^b^ |  |
| 2' |  | 111.4, CH | 7.57 ^c^ |  | 115.0, CH | 7.57, br s |  | 111.6, CH | 7.66, br s |
| 3' |  | 148.4, C |  |  | 146.2, C |  |  | 148.6, C |  |
| 4' |  | 150.6, C |  |  | 150.1, C |  |  | 151.4, C |  |
| 5' |  | 111.8, CH | 7.11, d (8.2) |  | 111.5, CH | 7.01, d (8.2) |  | 111.8, CH | 7.16, d (8.5) |
| 6' |  | 121.3, CH | 7.58 ^c^ |  | 120.1, CH | 7.49, br d (8.2) |  | 122.1, CH | 7.71, d (8.5) |
| 3-OMe |  | 59.7, CH_3_ | 3.77, s |  | 59.3, CH_3_ | 3.81, s |  | 59.8, CH_3_ | 3.83, s |
| 7-OMe |  |  |  |  | 55.5, CH_3_ | 3.80, s |  | 55.8, CH_3_ | 3.87, s ^c^ |
| 3'-OMe |  | 55.7, CH_3_ | 3.83, s |  |  |  |  | 55.8, CH_3_ | 3.86, s |
| 4'-OMe |  | 55.6, CH_3_ | 3.84, s |  | 55.4, CH_3_ | 3.88, s |  | 55.9, CH_3_ | 3.87, s ^c^ |

*^a^* ^13^C NMR data extracted from HSQC and HMBC spectra, *^b^* Signal not visible in HMBC, ^c^ Overlapping signals.

Table S 2. ^1^H and ^13^C NMR Spectroscopic Data for Compounds 4, 5 and 6 (DMSO-*d6*; 500.13 Hz for ^1^H and 125.77 for ^13^C NMR; δ in ppm)

|  |  | **4** | | | **5** | | **6** | | |
| --- | --- | --- | --- | --- | --- | --- | --- | --- | --- |
| Position |  | δ_C_*^a^* | δ_H_ (mult *J* in Hz*)* |  | δ_C_*^a^* | δ_H_ (mult *J* in Hz*)* |  | δ_C_*^a^* | δ_H_ (mult *J* in Hz*)* |
| 2 |  | 77.8, CH | 5.29, dd (12.2, 3.1) |  | 154.5, C |  |  | 155.9, C |  |
| 3 |  | 42.0, CH_2_ | 3.03, dd (16.9, 12.4)  2.56, dd (17.1, 3.1) |  | 137.7, C |  |  | 137.9, C |  |
| 4 |  | 192.8, C |  |  | 177.4, C |  |  | 178.0, C |  |
| 5 |  | 162.4, C |  |  | 161.0, C |  |  | 161.0, C |  |
| 6 |  | 96.8, CH | 5.59, d (1.5) |  | 99.0, CH | 6.14, d (1.8) |  | 97.5, CH | 6.22, s |
| 7 |  | 174.1, C |  |  | 166.4, C |  |  | 165.0, C |  |
| 8 |  | 97.4, CH | 5.57, d (1.5) |  | 93.7, CH | 6.34, d (1.5) |  | 92.0, CH | 6.50, br s |
| 9 |  | 163.7, C |  |  | 156.3, C |  |  | 156.1, C |  |
| 10 |  | 99.3, C |  |  | 103.2, C |  |  | 105.1, C |  |
| 1' |  | 129.5, C |  |  | 122.3, C |  |  | C*^b^* |  |
| 2' |  | 128.0, CH | 7.27, d (8.5) |  | 114.8, CH | 7.52, d (2.1) |  | 115.6, CH | 7.58, br s |
| 3' |  | 115.1, CH | 6.78, d (8.5) |  | 146.2, C |  |  | 145.3, C |  |
| 4' |  | 157.6, C |  |  | 149.9, C |  |  | 148.8, C |  |
| 5' |  | 115.1, CH | 6.78, d (8.5) |  | 111.8, CH | 7.05, d (8.5) |  | 115.7, CH | 6.92, br s |
| 6' |  | 128.0, CH | 7.27, d (8.5) |  | 119.9, CH | 7.51, dd (8.2, 2.1) |  | 120.6, CH | 7.43, br d (7.9) |
| 3-OMe |  |  |  |  | 59.4, CH_3_ | 3.78, s |  | 59.5, CH_3_ | 3.79, m |
| 7-OMe |  |  |  |  |  |  |  | 55.8, CH_3_ | 3.79, s |
| 4'-OMe |  |  |  |  | 55.4, CH_3_ | 3.85, s |  |  |  |

*^a^* ^13^C NMR data extracted from HSQC and HMBC spectra, *^b^* Signal not visible in HMBC.

Table S 3. ^1^H and ^13^C NMR Spectroscopic Data for Compound 7 (DMSO-*d6*; 500.13 Hz for ^1^H and 125.77 for ^13^C NMR; δ in ppm)

|  |  |  | **7** | |  |  | |
| --- | --- | --- | --- | --- | --- | --- | --- |
| Position |  |  | δ_C_*^a^* | δ_H_ (mult *J* in Hz*)* |  |  |  |
| 2 |  |  | 113.1, CH | 7.46 b |  |  |  |
| 3 |  |  | 147.0, C |  |  |  |  |
| 5 |  |  | 114.7, CH | 6.84, br s |  |  |  |
| 6 |  |  | 123.1, CH | 7.43 b |  |  |  |
| 3-OMe |  |  | 55.5, CH3 | 3.79, s |  |  |  |

*^a^* ^13^C NMR data extracted from HSQC and HMBC spectra, *^b^* Overlapping signals, ^c^ broad signal due to concentrated sample.

Table S 4. ^1^H and ^13^C NMR Spectroscopic Data for Compound **8** and **9** (DMSO-*d6*; 500.13 Hz for ^1^H and 125.77 for ^13^C NMR; δ in ppm)

|  |  |  | **8** | |  | **9** | |
| --- | --- | --- | --- | --- | --- | --- | --- |
| Position |  |  | δ_C_*^a^* | δ_H_ (mult *J* in Hz*)* |  | δ_C_*^a^* | δ_H_ (mult *J* in Hz*)* |
| 1 |  |  | 63.8, CH | 3.61, dd (5.8, 5.8) |  | 63.9, CH | 3.66 *^b^* |
| 1a |  |  | 39.6, CH_2_ | 2.94, dd (13.7, 6.1)  2.71 *^b^* |  | 39.8, CH_2_ | 2.98, dd (13.9, 6.0)  2.78, dd (13.9, 6.3) |
| 3 |  |  | 46.5, CH_2_ | 3.04, ddd (12.7, 8.0, 5.2)  2.56, m |  | 46.8, CH_2_ | 3.05, ddd (12.4, 7.8, 4.9)  2.59 ddd (12.2, 4.6, 4.6) |
| 4 |  |  | 24.9, CH_2_ | 2.67 *^b^*  2.47, m |  | 25.0, CH_2_ | 2.68, m  2.46, m |
| 4a |  |  | 126.1, C |  |  | 126.4, C |  |
| 5 |  |  | 111.7, CH | 6.59, s |  | 112.0, CH | 6.60, s |
| 6 |  |  | 146.8, C |  |  | 147.1, C |  |
| 7 |  |  | 146.1, C |  |  | 146.5, C |  |
| 8 |  |  | 111.5, CH | 6.32, s |  | 111.7, CH | 6.35, s |
| 8a |  |  | 129.5, C |  |  | 129.4, C |  |
| 1' |  |  | 132.9, C |  |  | 132.6, C |  |
| 2' |  |  | 114.0, CH | 6.62 *^b^* |  | 114.0, CH | 6.69, br s |
| 3' |  |  | 144.5, C |  |  | 148.4, C |  |
| 4' |  |  | 146.8, C |  |  | 147.1, C |  |
| 5' |  |  | 114.7, CH | 6.63 *^b^* |  | 111.9, CH | 6.79, d (8.2) |
| 6' |  |  | 121.8, CH | 6.51, dd (8.2, 1.2) |  | 121.8, CH | 6.64, d (7.6) |
| 2-NMe |  |  | 42.1, CH_3_ | 2.37, s |  | 42.4, CH_3_ | 2.38, s |
| 6-OMe |  |  | 55.3, CH_3_ | 3.68, s |  | 55.6, CH_3_ | 3.69, s |
| 7-OMe |  |  | 55.1, CH_3_ | 3.53, s |  | 55.4, CH_3_ | 3.54 *^b^* |
| 3'-OMe |  |  |  |  |  | 55.5, CH_3_ | 3.65, s *^b^* |
| 4'-OMe |  |  | 55.4, CH_3_ | 3.65, s |  | 55.7, CH_3_ | 3.70, s |

*^a^* ^13^C NMR data extracted from HSQC and HMBC spectra, *^b^* Overlapping signals.

Table S 5. ^1^H and ^13^C NMR Spectroscopic Data for Compound **10** and **11** (DMSO-*d6*; 500.13 Hz for ^1^H and 125.77 for ^13^C NMR; δ in ppm)

|  |  | **10** | |  | **11** | |
| --- | --- | --- | --- | --- | --- | --- |
| Position |  | δ_C_*^a^* | δ_H_ (mult *J* in Hz*)* |  | δ_C_*^a^* | δ_H_ (mult *J* in Hz*)* |
| 1 |  | 132.3, C |  |  |  |  |
| 2 |  | 110.6, CH | 6.92, br s |  | 146.1, C |  |
| 3 |  | 147.6, C |  |  |  |  |
| 4 |  | 146.0, C |  |  | 175.5, C |  |
| 5 |  | 115.3, CH | 6.77 *^b^* |  | 160.4, C |  |
| 6 |  | 118.6, CH | 6.77 *^b^* |  | 98.0, CH | 6.20, d (1.2) |
| 7 |  | 85.2, CH | 4.64, d (4.3) |  | 164.0, C |  |
| 8 |  | 53.6, CH | 3.05, m |  | 93.2, CH | 6.46, d (1.2) |
| 9 |  | 70.9, CH_2_ | 4.15, dd (8.90, 6.7)  3.76, dd (9.00, 3.5) |  | 155.8, C |  |
| 10 |  |  |  |  | 102.5, C |  |
| 1' |  | 132.3, C |  |  | 121.7, C |  |
| 2' |  | 110.6, CH | 6.92, br s |  | 111.7, CH | 7.77, br s |
| 3' |  | 147.6, C |  |  | 147.1, C |  |
| 4' |  | 146.0, C |  |  | 148.5, C |  |
| 5' |  | 115.3, CH | 6.77 *^b^* |  | 115.2, CH | 6.95, d (8.5) |
| 6' |  | 118.6, CH | 6.77 *^b^* |  | 121.3, CH | 7.69, br d (7.6) |
| 7' |  | 85.2, CH | 4.64, d (4.3) |  |  |  |
| 8' |  | 53.6, CH | 3.05, m |  |  |  |
| 9' |  | 70.9, CH_2_ | 4.15, dd (8.90, 6.7)  3.76, dd (9.00, 3.5) |  |  |  |
| 3-OMe |  | 55.7, CH_3_ | 3.78, s |  |  |  |
| 3'-OMe |  | 55.7, CH_3_ | 3.78, s |  | 55.5, CH_3_ | 3.85, s |

*^a^* ^13^C NMR data extracted from HSQC and HMBC spectra, *^b^* Overlapping signals.

Table S 6. ^1^H and ^13^C NMR Spectroscopic Data for Compound **12** (DMSO-*d6*; 500.13 Hz for ^1^H and 125.77 for ^13^C NMR; δ in ppm)

|  |  |  | **12** | |
| --- | --- | --- | --- | --- |
| Position |  |  | δ_C_*^a^* | δ_H_ (mult *J* in Hz*)* |
| 2 |  |  | 162.2, C |  |
| 3 |  |  | 107.2, CH | 6.92, s |
| 4 |  |  | 176.6, C |  |
| 5 |  |  | 128.0, CH | 7.97, d (8.5) |
| 6 |  |  | 108.9, CH | 7.00, d (8.5) |
| 7 |  |  | 153.7, C |  |
| 8 |  |  | 112.6, C |  |
| 9 |  |  | 164.3, C |  |
| 10 |  |  | 118.2, C |  |
| 1' |  |  | 131.3, C |  |
| 2' |  |  | 126.6, CH | 7.99 *^b^* |
| 3' |  |  | 129.4, CH | 7.55, m |
| 4' |  |  | 132.2, CH | 7.59, m |
| 5' |  |  | 129.4, CH | 7.55, m |
| 6' |  |  | 126.6, CH | 7.99 *^b^* |
| 2'' |  |  | 85.0, C |  |
| 3'' |  |  | 76.7, CH | 5.56, d (8.9) |
| 4'' |  |  | 47.8, CH | 4.85, dd (8.7, 6.6) |
| 5'' |  |  | 112.5, CH | 6.53, d (6.4) |
| 7'' |  |  | 27.5, CH_3_ | 1.33, s |
| 8'' |  |  | 23.3, CH_3_ | 1.05, s |
| 9'' |  |  | 169.4, C |  |
| 10'' |  |  | 20.3, CH_3_ | 1.42, s |

*^a^* ^13^C NMR data extracted from HSQC and HMBC spectra, *^b^* Overlapping signals.


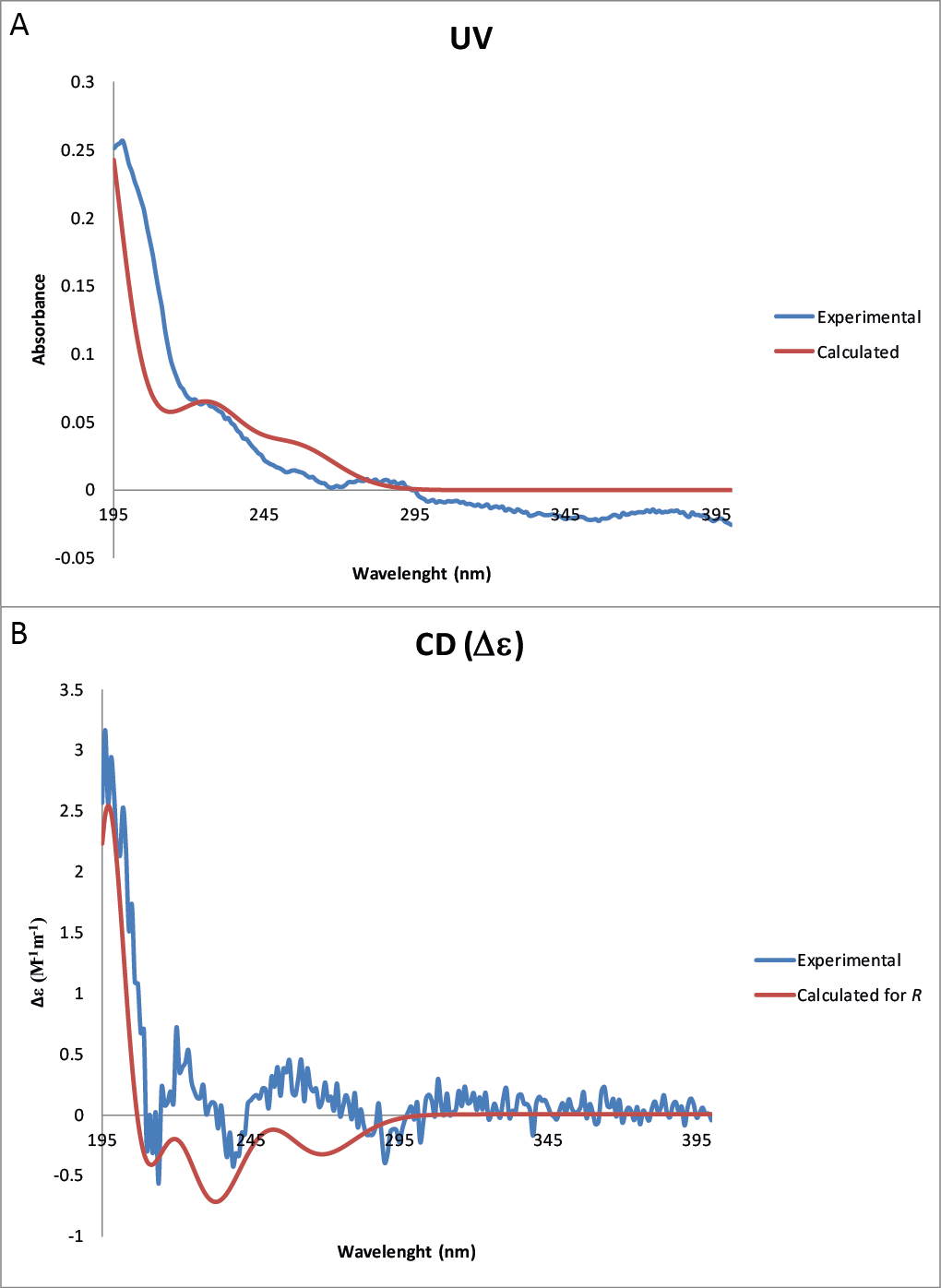


Figure S 1. Comparison of experimental and calculated UV (A) and ECD (B) spectra for compound 8 in MeOH (0.12 mg/mL).

Figure S 2. Comparison of experimental and calculated UV (A) and ECD (B) spectra for compound 9 in MeOH (0.05mg/mL).


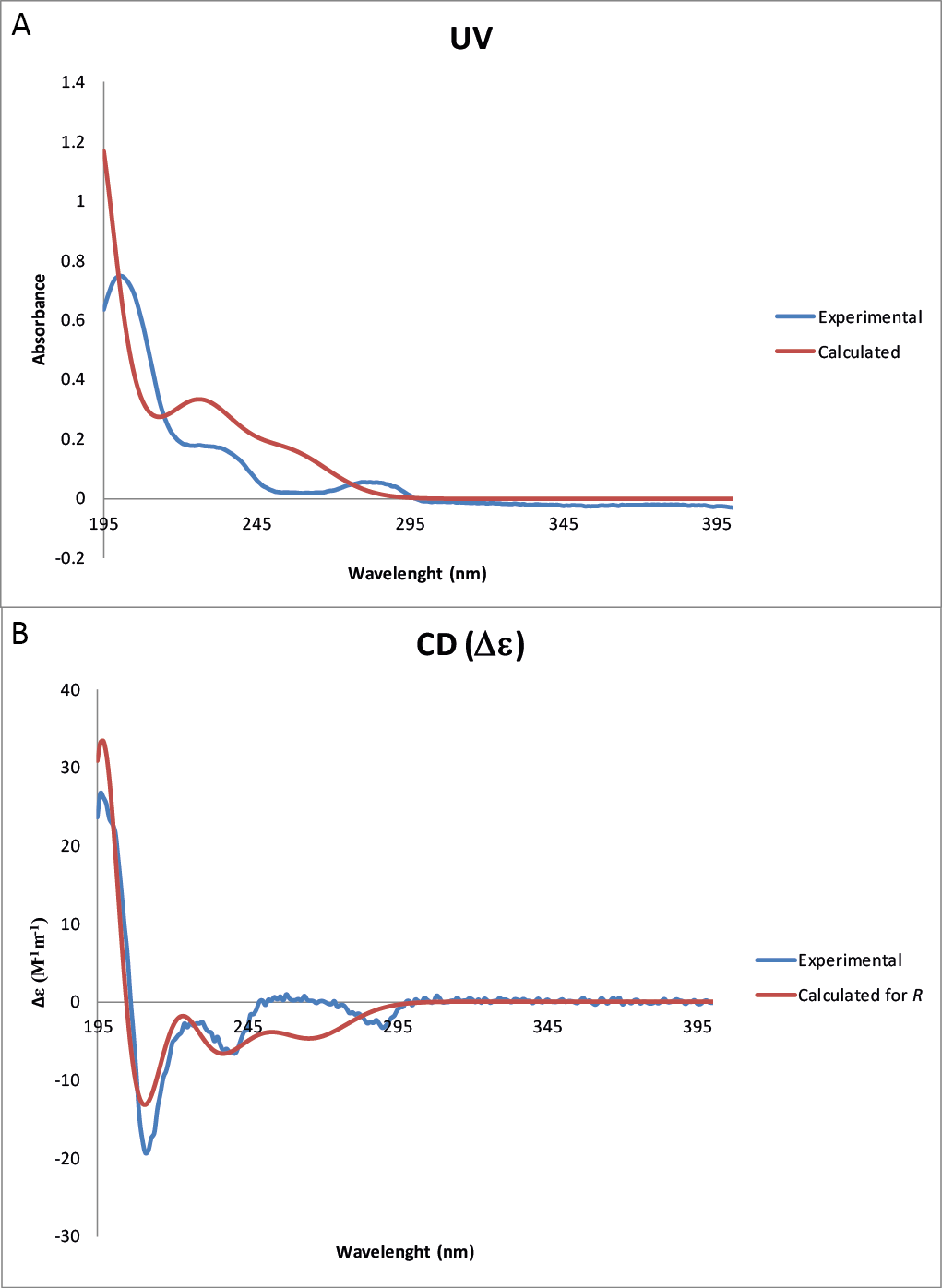


Figure S 3. Experimental UV (A) and ECD (B) spectra for compound 10 in MeOH (0.025 mg/mL).


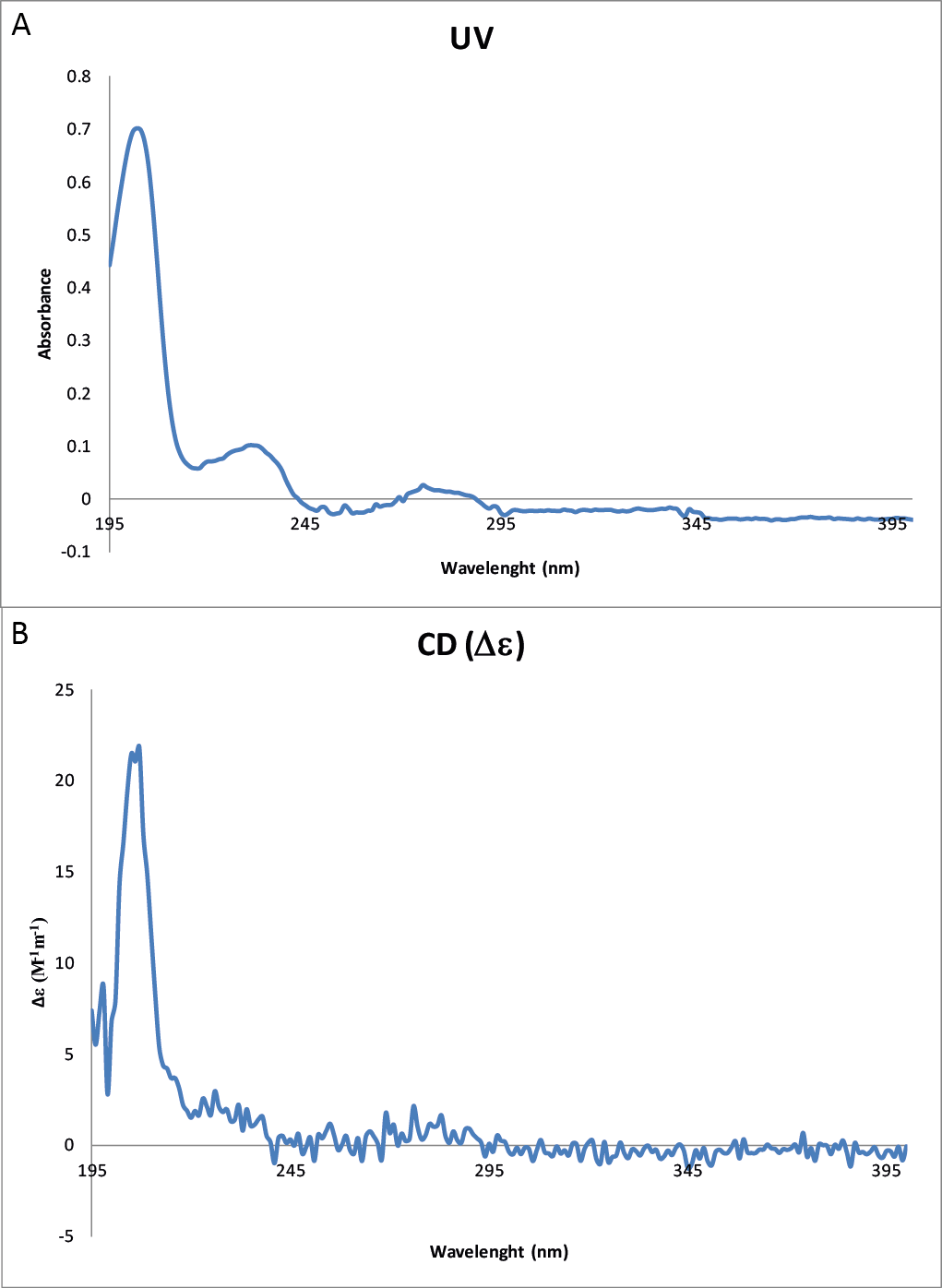


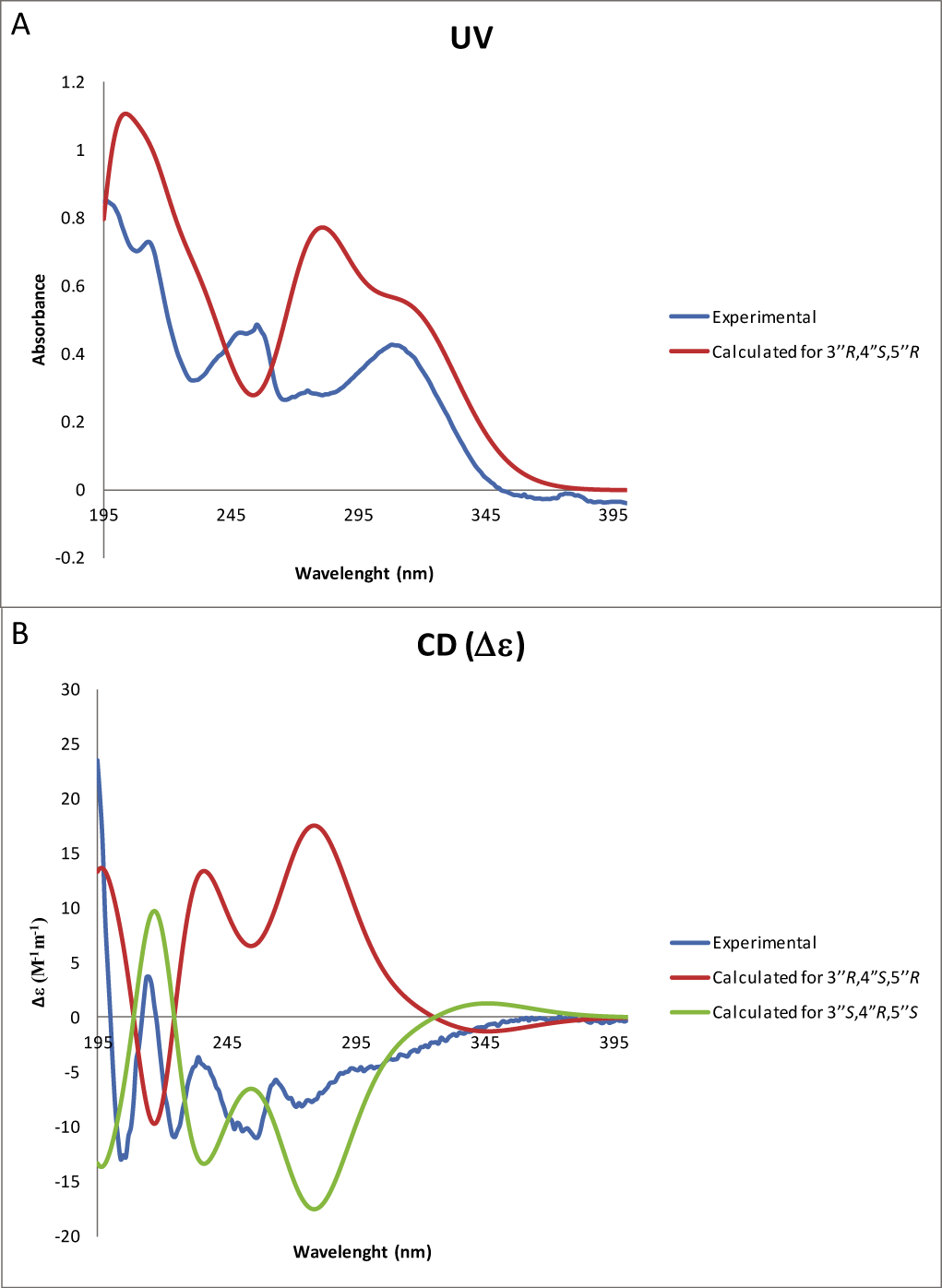
 Figure S 4. Comparison of experimental and calculated UV (A) and ECD (B) spectra for compound 12 in MeOH ( 0.1mg/mL).

**Computational Methods for ECD calculation.**

Conformational analysis of compounds **8**, **9** and 1**2** was performed with MacroModel 9.8 software (Schrödinger LLC) employing the OPLS 2005 (Optimized Potential for Liquid Simulations) force field in H_2_O. The five conformers with the lowest energy were submitted to geometrical optimization and energy calculation using Density Function Theory (DFT) with Becke’s nonlocal three-parameter exchange and correlation functional and the Lee-Yang-Parr correlation functional level (B3LYP) using the B3LYP/6-31+G(d,p) basis set in MeOH with the Gaussian 09 program package [1]. Vibrational evaluation was done at the same level to confirm minima. Excitation energy (denoted by wavelength in nm), rotator strength (Rstr), dipole velocity (Rvel), and dipole length (Rlen) were calculated in MeOH by TD-DFT/B3LYP/6- 31G(d,p). ECD curves were obtained on the basis of rotator strengths with a half-band of 0.3 eV using SpecDis v1.71 [2]. ECD spectra were calculated from the spectra of individual conformers according to their contribution calculated by Boltzmann weighting.

**References:**

^1^ Frisch MJ, Trucks GW, Schlegel HB, Becke AD. Gaussian 09, Revision A. 02, Gaussian, Inc., WalҮ lingford CT, 2009. J Chem Phys 1993; 98: 5648

^2^ Bruhn T, Hemberger Y, Schaumlöffel A, Bringmann G. SpecDis, Version 1.53; University of Wuerzburg: Wuerzburg, Germany, 2011. Received: July 2012; 2
